# Simultaneous and absolute quantification of nucleoside triphosphates using liquid chromatography–triple quadrupole tandem mass spectrometry

**DOI:** 10.1186/s41021-018-0101-8

**Published:** 2018-07-30

**Authors:** Shun Matsuda, Toshihiko Kasahara

**Affiliations:** Safety Evaluation Center, Ecology & Quality Management Division, CSR Division, FUJIFILM Corporation, 210 Nakanuma, Minamiashigara, Kanagawa 250-0193 Japan

**Keywords:** Nucleoside triphosphates, Multiple reaction monitoring/selected reaction monitoring (MRM/SRM), Absolute quantification, Thymidine, Molm-13 cells

## Abstract

**Background:**

Nucleoside triphosphates participate in fundamental cellular processes as building blocks of DNA and RNA, energy carriers, and cofactors in enzymatic reactions, and their balance is tightly regulated. Here, we established a simultaneous and absolute quantification method for eight nucleoside triphosphates using liquid chromatography–triple quadrupole tandem mass spectrometry and hydrophilic interaction chromatography. Our method was successfully applied to the extract of human acute myeloid leukemia Molm-13 cells.

**Results:**

Levels of ribonucleoside triphosphates (2.07 × 10^8^–2.29 × 10^9^ molecules/cell) in Molm-13 cells were two orders of magnitude higher than those of deoxyribonucleoside triphosphates (1.72 × 10^6^–1.40 × 10^7^ molecules/cell). Exposure of Molm-13 cells for 24 h to thymidine, a nucleotide imbalance inducer, increased the levels of cellular dTTP, dGTP, and dATP and decreased only dCTP, resulting in significant inhibition of cell proliferation.

**Conclusion:**

Our quantification method for nucleoside triphosphates revealed the quantitative relationship between the arrest of cell proliferation and the imbalance of nucleoside triphosphates in thymidine-treated Molm-13 cells. Owing to the short run time (15 min/run), broad adaptability, and throughput performance, we believe that our method is a powerful tool for not only genetic and molecular biology research but also for studying the mechanism of genotoxic compounds and anti-cancer or anti-virus drugs, drug screening, clinical studies, and other fields.

## Background

Nucleoside triphosphates are building blocks of DNA and RNA, donors of phosphate groups, energy carriers, cofactors in enzymatic reactions, and participate in cellular signaling [1, 2]. Thus, cells have elegant systems to tightly regulate the balance of the levels of these metabolites, such as a negative feedback system of nucleotide biosynthesis [1] and the selective production of deoxyribonucleotides by ribonucleotide reductase (RNR) [3]. Imbalance of cellular nucleoside triphosphates have harmful effects to cells, including inhibition of gene transcription [4] and protein translation [5] and induction of DNA mutation, which is one of the major factors of carcinogenesis [6, 7]. In fact, defects in nucleotide biosynthesis are responsible for some genetic diseases such as Lesch-Nyhan syndrome, mitochondrial depletion syndrome, and mitochondrial neurogastrointestinal encephalomyopathy [8]. Moreover, nucleotide biosynthesis is an attractive target of drug discovery in the field of pharmacology. Metabolic antagonists, represented by 5-fluorouracil and hydroxyurea, inhibit nucleotide biosynthesis to kill cancer cells; thus, these types of drugs are continuously being developed [9]. Furthermore, various types of nucleoside and nucleotide analogues (including 5-fluorouracil), which have been developed as anti-cancer and anti-virus drugs [10], can induce an imbalance of cellular nucleoside triphosphates as the main effect or side effects. Therefore, analysis of cellular nucleoside triphosphates is useful not only for genetic and molecular biology research but also for mechanistic studies of disease, drugs, and genotoxic compounds, and for evaluation of the efficacy and side effects of drugs.

Some excellent methods for the simultaneous quantification of cellular nucleoside triphosphates using liquid chromatography-tandem mass spectrometry (LC-MS/MS) have been reported. Chen et al. [11] established a simultaneous quantification method for GTP, CTP, UTP, dATP, dCTP, and dTTP; however, this method could not separate ATP and dGTP in either liquid chromatography or mass spectrometry. Zhang et al. and Kamceva et al. reported a method for the simultaneous quantification of eight nucleoside triphosphates, including ATP and dGTP [12, 13]. However, these methods still have some methodological limitations. First, these methods use a cellular matrix in samples for generating calibration curves or a standard addition method to correct for a matrix effect. The former requires a burdensome process to remove analytes (nucleotides) in the cell extract. The process could also produce artifacts in the cellular matrix, and the cellular matrix sample used for calibration curves must be prepared with respect to each cell line of interest because the composition of the cellular matrix may differ among cell lines. The latter requires preparation of calibration curves with respect to each sample. These processing steps are limitations of high-throughput analysis. Second, the run time of LC-MS/MS analysis in these quantification methods is relatively long (from 40 min to over 100 min). Third, all of these quantification methods use ion-pair reagents, which persistently remain in the flow path in LC-MS/MS and can cause adverse effect for the analysis of other analytes not requiring ion-pair agents, such as a change of retention time and peak shape, and ion suppression. On the other hand, a few ion-pair agent-free quantification methods of nucleoside triphosphates using LC-MS/MS were reported. Thomas et al. established a simultaneous quantification method of nucleoside triphosphates using anion exchange chromatography [14]. The method could overcome the limitations which ion-pair agent-dependent methods have, but uses a standard addition method, which has limitations of high-throughput analysis as described above. Nakamura et al. reported a quantification method of ATP using hydrophilic interaction chromatography (HILIC) [15]. HILIC has a good separation performance against highly hydrophilic analytes (nucleoside triphosohates are also highly hydrophilic) and is compatible with MS.

Based on HILIC, in this study, we developed a simple, ion-pair agent-free, and reliable method for the absolute quantification of nucleoside triphosphates using their isotope-labeled internal standards and LC-MS/MS. Furthermore, we successfully applied this method to human cell samples, and captured the physiological change of these metabolite levels in cells after treatment with thymidine, a nucleotide imbalance inducer.

## Methods

### Materials

RPMI 1640 medium, penicillin, streptomycin, and fetal bovine serum (FBS) were purchased from Life Technologies (Carlsbad, CA, USA). dATP was purchased from GE Healthcare (Chicago, IL, USA). dGTP, dCTP, and dTTP were purchased from Thermo Fisher Scientific (Waltham, MA, USA). [^15^N_5_,^13^C_10_]ATP, [^15^N_5_,^13^C_10_]GTP, [^15^N_3_,^13^C_9_]CTP, [^15^N_2_,^13^C_9_]UTP, [^15^N_5_,^13^C_10_]dATP, [^15^N_5_,^13^C_10_]dGTP, [^15^N_3_,^13^C_9_]dCTP, and [^15^N_2_,^13^C_10_]dTTP were purchased from Sigma-Aldrich (St. Louis, MO, USA). The other chemicals were purchased from Wako (Osaka, Japan). A stock solution of thymidine was dissolved in ultrapure water.

### Cell culture

Human acute myeloid leukemia Molm-13 cells were obtained from DSMZ (Braunschweig, Germany), and were cultured in RPMI 1640 medium supplemented with 10% FBS, 100 units/ml penicillin, and 100 μg/ml streptomycin at 37 °C in a humidified 5% CO_2_ incubator.

### Metabolite extraction

Molm-13 cells (1 × 10^6^ cells in 2 ml of the medium) were treated with vehicle [2% (*v*/v) ultrapure water] or thymidine for 24 h. The chemical solution was directly added to the medium. The cell suspension was transferred to a 15-ml tube, and 150 μl of the suspension was saved for cell counting to determine cell survival. The remaining cell suspension was pelleted by centrifugation at 1000 rpm for 5 min using an LC-200 centrifuge (Tomy, Tokyo, Japan), and the supernatant was removed by decantation. After addition of 10 ml of ice-cold phosphate buffered saline (PBS), the cell pellet was resuspended and pelleted again by centrifugation at 1000 rpm for 5 min. After the supernatant was removed by decantation, 1 ml of ice-cold PBS was added to the cell pellet and resuspended. One hundred microliters of the suspension was saved for cell counting to determine the cell number to be used for metabolite extraction. One milliliter of the remaining suspension was transferred into a 1.5-ml tube, pelleted by centrifugation at 2000×*g* for 2 min at 4 °C using a centrifuge MX-301 (Tomy), and 900 μl of the supernatant was removed. To extract metabolites, 500 μl of methanol was added to the remaining sample and vortexed vigorously. Furthermore, 10 μl of the internal standard mix (10 μM [^15^N_5_,^13^C_10_]ATP, 10 μM [^15^N_5_,^13^C_10_]GTP, 10 μM [^15^N_3_,^13^C_9_]CTP, 10 μM [^15^N_2_,^13^C_9_]UTP, 10 μM [^15^N_5_,^13^C_10_]dATP, 10 μM [^15^N_5_,^13^C_10_]dGTP, 10 μM [^15^N_3_,^13^C_9_]dCTP, and 10 μM [^15^N_2_,^13^C_10_]dTTP) and 190 μl of ultrapure water were added to the sample and vortexed vigorously. After centrifugation at 10,000×*g* for 15 min at 4 °C, 700 μl of the supernatant was transferred to a new 1.5-ml tube, and evaporated to dryness at 37 °C using a centrifugal evaporator (CVE-3100; Tokyo Rikakikai, Tokyo, Japan). The dried sample was stored at −20°C until the time of use. Cell counting was performed using a Coulter counter Z2 system (Beckman Coulter, CA, USA).

### LC-MS/MS

The dried sample was redissolved in 100 μl of a mixture of 10 mM ammonium bicarbonate and acetonitrile [35 (v):65 (v)]. After centrifugation at 16,000×*g* for 5 min, 70 μl of the supernatant was transferred to a vial. Mass spectrometric analysis was performed using a Xevo TQ-S micro mass spectrometer (Waters, Manchester, UK) with an H-classBio system (Waters). Two microliters of each sample was separated on a SeQuant ZIC-pHILIC HPLC column (5 μm, 4.6 × 150 mm; Merck Millipore, Darmstadt, Germany) at a flow rate of 0.5 ml/min, and subsequently eluted as follows: solvent A, 10 mM ammonium bicarbonate and 0.05% ammonium hydroxide; solvent B, acetonitrile: 0–5 min, linear gradient from 65% B to 40% B; 5–7 min, linear gradient to 0% B; 7–9 min, isocratic with 0% B; 9–9.1 min, linear gradient to 65% B; 9.1–15 min, isocratic with 65% B. Multiple reaction monitoring (MRM) was performed in positive-ion mode using nitrogen as the nebulizing gas. Experimental conditions were set as follows: ion source temperature, 150 °C; desolvation temperature, 550 °C; desolvation gas flow rate, 1200 l/h; capillary voltage, 2.0 kV; cone gas flow rate, 110 l/h; collision gas, argon. The conditions of the MRM transitions were as follows [cone voltage (V), collision energy (eV)]: ATP, 508.1 > 136.0 (20, 35); GTP, 524.1 > 152.0 (20, 25); CTP, 484.1 > 112.0 (20, 20); UTP, 485.0 > 96.9 (20, 25); dATP, 492.1 > 136.0 (20, 20); dGTP, 508.1 > 152.0 (20, 20); dCTP, 468.0 > 112.0 (20, 15); dTTP, 483.0 > 81.0 (50, 15); [^15^N_5_,^13^C_10_]ATP, 523.1 > 146.0 (20, 35); [^15^N_5_,^13^C_10_]GTP, 539.1 > 162.1 (20, 25); [^15^N_3_,^13^C_9_]CTP, 496.1 > 119.0 (20, 20); [^15^N_2_,^13^C_9_]UTP, 496.0 > 101.9 (20, 25); [^15^N_5_,^13^C_10_]dATP, 507.1 > 146.0 (20, 20); [^15^N_5_,^13^C_10_]dGTP, 523.1 > 162.1 (20, 20); [^15^N_3_,^13^C_9_]dCTP, 480.1 > 119.0 (20, 15); [^15^N_2_,^13^C_10_]dTTP, 495.1 > 86.0 (50, 15). The amount of each metabolite was quantified by calculating the peak area ratio of the target metabolite and its isotope-labeled internal standard. The calibration curve was obtained using an authentic standard metabolite spiked with its isotope-labeled internal standard.

## Results

### Method development

We used eight standard metabolites (dATP, dGTP, dCTP, dTTP, ATP, GTP, CTP, and UTP) and their respective isotope-labeled metabolites as internal standards. The use of isotope-labeled standards is one of the key advantages of our method, because they have identical separation, ionization, and fragmentation patterns, which results in high precision and accuracy [16]. MRM transitions for these metabolites were manually optimized. As cellular dNTP levels are reported to be about two orders of magnitude lower than cellular NTP levels [1], we obtained calibration curves at different ranges of concentrations between dNTPs and NTPs. As a result, calibration curves were linear over the entire range of 3–1000 nM for dNTPs and 300–100,000 nM for NTPs. The correlation coefficients were 0.9983 for dATP, 0.9977 for dGTP, 0.9990 for dCTP, 0.9981 for dTTP, 0.9995 for ATP, 0.9994 for GTP, 0.9997 for CTP, and 0.9996 for UTP. Next, the precision and accuracy were calculated at three levels for each metabolite. The obtained precision and accuracy based on eight replicate experiments ranged from 0.6 to 5.4% CV and from −3.1 to 2.5% Bias for all metabolites (Table 1). These data indicate that our method has acceptable accuracy and precision.

**Table 1:** Accuracy and precision data (*n* = 8)

Analyte	Expected concentration (nM)	Calculated concentration (nM)	%CV	%Bias
dATP	10	10.0	4.0	0.4
	100	100.9	1.2	0.9
	1000	987.8	1.4	−1.2
dGTP	10	10.1	5.4	1.2
	100	100.4	3.3	0.4
	1000	969.3	2.9	−3.1
dCTP	10	9.8	3.3	−1.6
	100	102.0	0.6	2.0
	1000	1000.5	1.6	0.1
dTTP	10	10.2	2.0	2.0
	100	99.5	1.5	−0.5
	1000	983.8	3.2	−1.6
ATP	1000	1004.5	2.0	0.5
	10,000	10,068.8	0.9	0.7
	100,000	98,628.5	1.4	−1.4
GTP	1000	1004.3	1.1	0.4
	10,000	10,197.3	2.0	2.0
	100,000	98,085.3	1.9	−1.9
CTP	1000	1002.0	1.1	0.2
	10,000	10,060.3	1.4	0.6
	100,000	98,854.1	0.8	−1.1
UTP	1000	1006.3	2.3	0.6
	10,000	10,252.5	2.8	2.5
	100,000	100,137.9	2.5	0.1

### Application to extract from cells

**Fig. 1:**
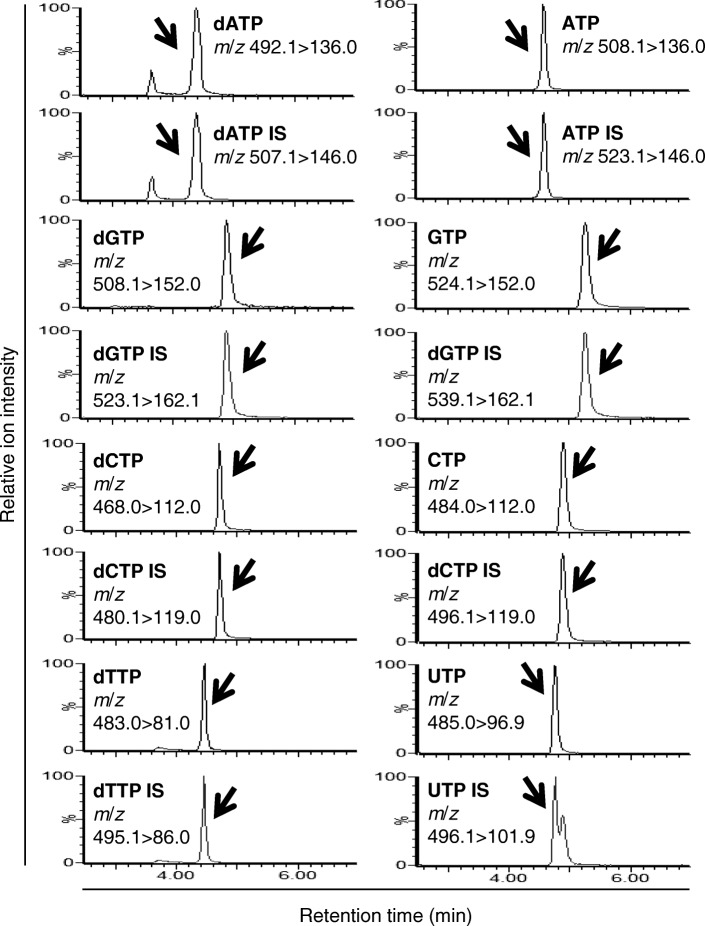
MS/MS chromatograms of the analytes and their internal standards (IS) in the extract from Molm-13 cells. Peaks representing analytes or IS are indicated by arrows

We tested whether our method can be applied to a biological sample. Immediately after metabolite extraction from Molm-13 cells by methanol, the isotope-labeled internal standards of dNTPs and NTPs were spiked to the extract to correct for variation between samples produced by the subsequent handling steps. After evaporation to dryness and re-dissolution, we applied our method to try to detect dNTPs and NTPs. As shown in Fig. 1, obvious peaks representing dNTPs and NTPs were observed at the same retention times as their respective internal standards. These data confirmed that our method is able to specifically detect these metabolites from cell samples.

### Quantitative analysis of cell samples

**Fig. 2:**
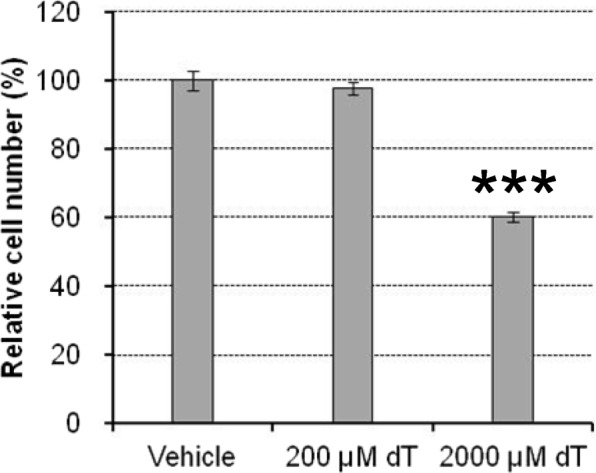
Inhibition of cell proliferation of Molm-13 cells treated with 200 or 2000 μM thymidine (dT) for 24 h. Mean ± SD, *n* = 3. ***p < 0.001 versus vehicle (*t*-test)

Next, we attempted to quantify the absolute amounts of dNTPs and NTPs in the extract from Molm-13 cells. Thymidine was used as a nucleotide pool disruptor for this purpose. Molm-13 cells were treated with thymidine at two concentrations: the lower concentration (200 μM) did not significantly affect the proliferation of cells, whereas the higher concentration inhibited the proliferation to 60% that of the vehicle control group (Fig. 2). After treatment, we determined the cell number to be used for metabolite extraction as described in the Materials and Methods section, and calculated the number of molecules of each metabolite in a Molm-13 cell according to the following equation:

$$ Molecules/ cell\cong \frac{Metabolite\ amount\kern0.5em (mol)}{Cell\ number}\times 6.02\times {10}^{23}\ \left( Avogadro\ constant\right) $$

In the vehicle control condition, the amounts of dNTPs in a cell were calculated as 4.63 × 10^6^ molecules for dATP, 1.72 × 10^6^ molecules for dGTP, 4.22 × 10^6^ molecules for dCTP, and 1.40 × 10^7^ molecules for dTTP. Thymidine increased the cellular dTTP level in a dose-dependent manner, which showed the most drastic increase of 12.9-fold (1.81 × 10^8^ molecules/cell) after treatment with 2000 μM thymidine. Inversely, thymidine decreased the cellular dCTP level in a dose-dependent manner (2.02 × 10^6^ molecules/cell at 2000 μM thymidine). The level of cellular dGTP was not affected by treatment of 200 μM thymidine, while 2000 μM thymidine increased the dGTP level by 4.6-fold (7.83 × 10^6^ molecules/cell). The dATP level was decreased by 0.69-fold (3.19 × 10^6^ molecules/cell) after treatment with 200 μM thymidine, but increased by 1.41-fold (6.52 × 10^6^ molecules/cell) after 2000 μM thymidine treatment. The amounts of NTPs in a cell were one order of magnitude or more than those of dNTPs: 2.29 × 10^9^ molecules for ATP, 5.05 × 10^8^ molecules for GTP, 2.07 × 10^8^ molecules for CTP, and 5.20 × 10^8^ molecules for UTP. The CTP level was increased by thymidine treatment in a dose-dependent manner, and it was increased by 1.56-fold (3.22 × 10^8^ molecules/cell). The levels of GTP and UTP were not changed by 200 μM thymidine treatment, while 2000 μM thymidine increased these levels (1.21-fold for GTP and 2.02-fold for UTP). The cellular ATP level showed no change under our experimental conditions.

## Discussion

We established a simple and reliable method for the simultaneous and absolute quantification of eight nucleoside triphosphates using stable isotope-labeled standards, HILIC, and LC-MS/MS. Moreover, using this method, we successfully captured changes in the cellular levels of these metabolites after thymidine treatment.

**Fig. 3:**
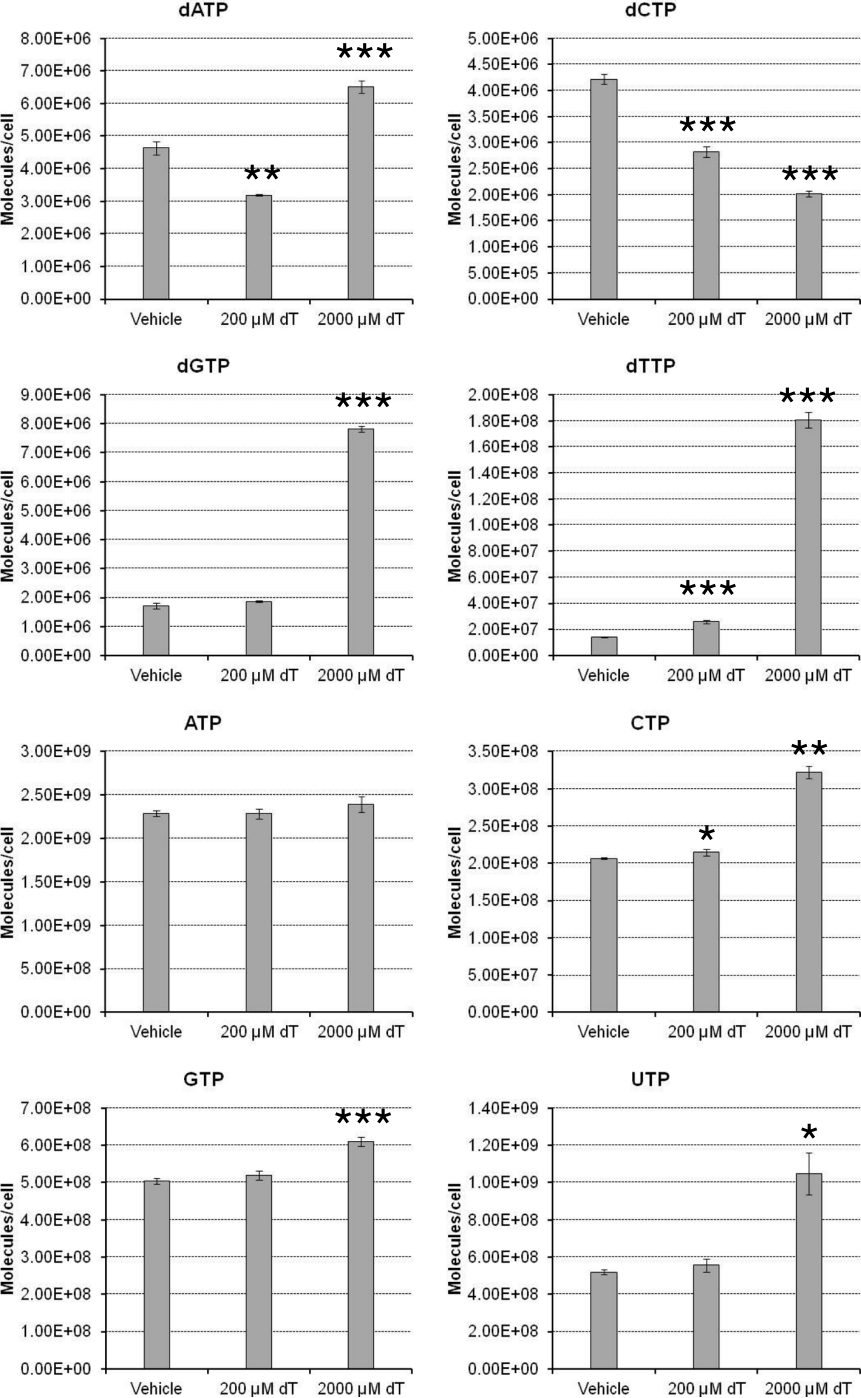
Change in dNTP and NTP levels in Molm-13 cells treated with 200 or 2000 μM thymidine (dT) for 24 h. Mean ± SD, *n* = 3. *p < 0.05, **p < 0.01, ***p < 0.001 versus vehicle (*t*-test)

**Fig. 4:**
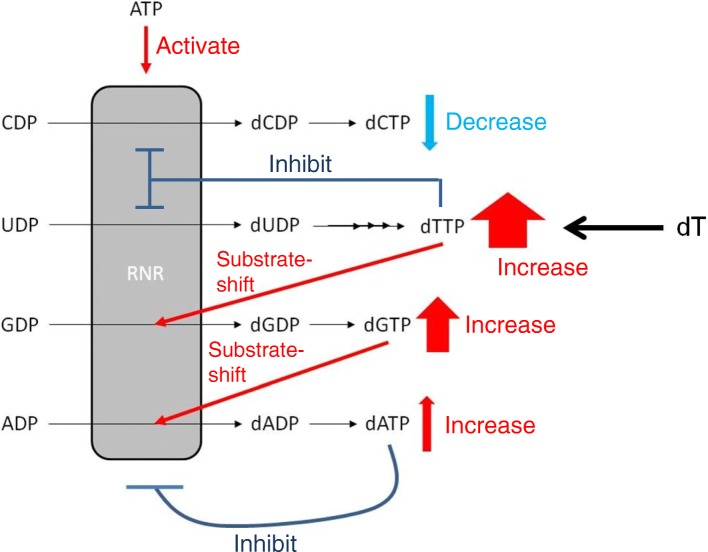
Possible mechanism of change in dNTP levels in Molm-13 cells induced by 2000 μM thymidine treatment for 24 h. RNR, ribonucleotide reductase; dT, thymidine. The width of the arrows indicating “increase” reflects the amount of change in the dNTP induced by thymidine treatment

As thymidine is converted to dTTP through a pyrimidine salvage pathway in cells, excess thymidine treatment drastically increases the cellular dTTP level. That induces a nucleotide imbalance, which activates the cell cycle checkpoint, resulting in the arrest of proliferation. In cell biology research, this property of thymidine is used for synchronization of the cell cycle (well known as the “double thymidine block”) [17]. However, the precise mechanism by which excess thymidine results in the loss of balance of nucleotides in a cell is largely unknown. In this study, exposure of Molm-13 cells to 2000 μM thymidine, which caused significant inhibition of cell proliferation (Fig. 2), increased not only dTTP but also dGTP and dATP levels, but decreased only the dCTP level (Fig. 3). The ratios of the cellular dNTP levels in the thymidine treatment group compared to those of the vehicle condition were 12.9-fold for dTTP, 4.6-fold for dGTP, 1.41-fold for dATP, and 0.48-fold for dCTP. This result could be explained by regulation of deoxyribonucleotide production by RNR (Fig. 4). RNR reduces NDPs to produce dNDPs, which are subsequently phosphorylated to dNTPs in cells (dTTP is produced from dUDP through several biochemical reactions). The activity and substrate selectivity of RNR are allosterically regulated by its substrates, products, and ATP [3]. In a condition of excess dTTP, which was induced by exposure of Molm-13 cells to excess thymidine, the dTTP-bound RNR suppresses reduction of CDP and UDP and preferentially reduces GDP to produce dGDP, which is then phosphorylated to produce dGTP in cells. The resulting elevation of cellular dGTP promotes dGTP binding to RNR, which prefers reduction of ADP to dADP. In turn, dATP, produced by phosphorylation of dADP in cells, binds to the activity regulation site of RNR to inhibit RNR activity. In this situation, the cells consume dNTPs for proliferation, thereby inducing the depletion of only cellular dCTP, resulting in cell proliferation arrest (Fig. 2). On the other hand, contrary to the case of 2000 μM thymidine treatment, the dATP level was decreased in Molm-13 cells treated with low concentration (200 μM) of thymidine, which did not affect proliferation of the cells (Figs. 2 and 3). This phenomenon may be difficult to be explained only using regulation by RNR. The relatively slight increase in the level of dTTP in the 200 μM thymidine-treated Mom-13 cells may suppress reduction of CDP by RNR, but may be insufficient to significantly increase the levels of dGTP (Fig. 3). For this reason, increase in the levels of the cellular dATP by substrate switching of RNR seems not to be observed. To rescue the decrease of the cellular dCTP level induced by thymidine, mechanisms other than RNR, such as adenosine deaminase which negatively regulates dATP level [18], may contribute to the decrease of the cellular dATP level that upregulates enzymatic activity of RNR. Our method would be useful for mechanistic analysis of genotoxic compounds and drugs that interrupt the cellular nucleotide balance.

Previous reports demonstrated that nucleotide imbalance activates the cell cycle checkpoint to arrest the cell cycle at the G_0_/G_1_ phase [19–21]. However, the quantitative relationship between this nucleotide imbalance and cell cycle arrest is largely unknown. The number of dNTP molecules required for replication to produce a human diploid genome can be calculated according to the following formula:

$$ dNTP\ molecules\cong human\ genome\ size\ (bp)\times 2\ (strands)\times 2\ (ploidy)\times \kern0.5em X\ \left(\%\right) $$

Here, the human genome size is 3.26 × 10^9^ bp according to Genome Reference Consortium Human Build 38 patch release 12, and *X* is the base composition of human DNA (29.3% A, 20.7% G, 20.0% C, and 30.0% T) [22]. Following the equation above, the number of dNTP molecules required for replication to produce a human diploid genome is calculated at 3.82 × 10^9^ for dATP, 2.70 × 10^9^ for dGTP, 2.61 × 10^9^ for dCTP, and 3.91 × 10^9^ for dTTP. These predicted values were compared with the result of dNTP molecules in a Molm-13 cell quantified in this study (Fig. 3), as shown in Table 2. In the vehicle condition, there was an extremely lower number of dNTP molecules in a Molm-13 cell than required for replication to produce a human diploid genome (0.064–0.358%). One of the plausible reasons is that cells may maintain cellular dNTPs at extremely low levels to suppress any unnecessary DNA replication, which could cause mutations, so as to maintain genome integrity. Although the decrease of cellular dCTP by treatment of 200 μM thymidine reaching a C base proportion of 0.109% in a human diploid genome did not affect cell proliferation, treatment with 2000 μM thymidine significantly inhibited cell proliferation, in which the cellular dCTP content decreased to 0.077% of C bases in a human diploid genome (Fig. 2 and Table 2). This result indicates that the threshold cellular dCTP level for activation of the cell cycle checkpoint in Molm-13 cells is in the range of 0.077–0.109% of C bases in a human diploid genome (2.02–2.83 × 10^6^ molecules/cell). It is noted that the quantification results in this study reflect the “average” values of cells in each condition, and that variability of nucleoside triphosphate levels among cells was not considered. Nonetheless, our method is expected to help promote a quantitative understanding of molecular biology and further contribute to research in other fields.

**Table 2:** Comparison of dNTP molecules in a Molm-13 cell with those required to build a diploid genome

			Ratio of cellular dNTP molecules to those required to build a diploid genome (%)		
Base	Substrate	Molecule number required to build a diploid genome^a^	Vehicle (100%^b^)	200 μM Thymidine (100% ^b^)	2000 μM Thymidine (60%^b^)
A	dATP	3.82 × 10^9^	0.121	0.083	0.171
G	dGTP	2.70 × 10^9^	0.064	0.069	0.290
C	dCTP	2.61 × 10^9^	0.162	0.109	0.077
T	dTTP	3.91 × 10^9^	0.358	0.662	4.627

^a^Information of human genome size and composition of each base in the genome was obtained from Genome Reference Consortium Human Build 38 patch release 12 and [22], respectively

^b^Relative cell number in each condition as shown in Fig. 2

## Conclusion

We developed an LC-MS/MS-based method for the absolute quantification of dNTPs and NTPs. This method could accurately and precisely quantitate these metabolites at ranges of 3–1000 nM for dNTPs and 300–100,000 nM for NTPs. We successfully applied this method to human cell samples. Owing to its short run time (15 min/run), broad adaptability, and throughput performance, we believe that our method is a powerful tool for not only genetic and molecular biology research but also for studying the mechanism of genotoxic compounds and drugs, anti-cancer and anti-virus drug screening, clinical studies, and other fields.

## Abbreviations

FBS: Fetal bovine serum
HILIC: Hydrophilic interaction chromatography
LC-MS/MS: Liquid chromatography-tandem mass spectrometry
MRM: Multiple reaction monitoring
PBS: Phosphate buffered saline
